# Supplementary material for: Yeast Diversity Associated with the Phylloplane of Corn Plants Cultivated in Thailand
Source: Microorganisms. 2020 Jan 7;8(1):80. doi: 10.3390/microorganisms8010080 (PMC7022409; doi:10.3390/microorganisms8010080)
Supplement: Supplementary file 1 [file microorganisms-08-00080-s001.pdf]

Table S1. Yeast species and isolates from corn leaf samples in each province.

Taxa	Strain DMKU-			
	Suphan Buri	Chainat	Nakhon Sawan	Phetchabun
**Phylum Ascomycota**				
**Subphylum Saccharomycotina**				
Candida intermedia	CP563	CP635,	CP669, CP705, CP791	-
Candida quercitrusa	-	-	-	CP719
Candida saopaulonensis	-	CP793, CP802	-	CP708, CP715
Candida uthaithanina	-	CP825	-	-
Meyerozyma caribbica	CP566, CP628	CP629, CP649, CP813	CP666, CP670, CP679, CP696, CP783	CP710, CP724, CP749, CP767,
Kodamaea ohmeri	CP586	-	-	-
Metschnikowia lopburiensis	-	CP816	CP680	CP722
Pichia bruneiensis	-	CP805, CP826	-	CP733, CP737, CP753, CP761
Pichia kudriavzevii	CP565		CP672	
Wickerhamomyces anomalus	CP571, CP577	CP657	-	-
**Phylum Basidiomycota**				
**Subphylum Agaricomycotina**				
Dioszegia zsoltii	CP555	CP647	CP780	-
Hannaella oryzae	-	-	CP701, CP775	-
Hannaella pagnoccae	-	CP636, CP810	-	CP723
Hannaella phetchabunensis	-	CP812	-	-
Hannaella phyllophila	-	CP648	CP667	CP763
Hannaella siamensis	CP590	CP631, CP822	-	CP721, CP743
Hannaella sinensis	CP553, CP568, CP595, CP606, CP610	CP630, CP637, CP643, CP652, CP655, CP792, CP803, CP819	CP665, CP668, CP677, CP690, CP703, CP787,	CP712, CP716, CP728, CP742, CP765
Vishiacozyma aff. *heimaeyensis*	CP576	CP796	-	-
Kwoniella bestiolae	CP619	-	-	-
Kwoniella heveanensis	-	CP820	-	-
Papiliotrema flavescens	CP611	CP634, CP642, CP654, CP806, CP808	CP700, CP786	CP714, CP720, CP734, CP740, CP760, CP768
Papiliotrema laurentii	CP560, CP567, CP585	CP797	CP660	-

Table S1. *Cont*

Taxa	Strain DMKU-			
	Suphan Buri	Chainat	Nakhon Sawan	Phetchabun
Papiliotrema rajasthanensis	CP552, CP580, CP592, CP603, CP621	CP800, CP823	CP691, CP776, CP790	CP754
Papiliotrema ruineniae	-	CP818	CP781	-
Plapiliotrema plantarum	-	CP801	-	-
Saitozyma flava	CP620	-	-	CP758
**Subphylum Pucciniomycotina**				
Cystobasidium calyptogenae	CP557	-	-	-
Symmetrospora vermiculata	CP558	CP821	CP771	-
Rhodotorula mucilaginosa	CP573, CP596	-	-	-
Rhodotorula paludigena	CP572, CP591, CP604, CP614, CP625	CP640, CP645, CP650, CP794	CP659, CP693, CP702, CP777, CP785	CP730, CP746, CP752, CP757
Rhodotorula toruloides	-	-	CP699	-
Rhodotorula aff. *toruloides*	CP570	CP639	-	-
Rhodosporidiobolus nylandii	CP589	-	-	-
Rhodosporidiobolus ruineniae	-	-	CP694, CP706	CP713
Rhodosporidiobolus fluvialis	-	CP658	-	-
Rhodosporidiobolus odoratus	CP622	-	-	-
Sporobolomyces blumeae	CP551, CP575, CP601, CP605, CP613	-	CP683, CP779	-
Sporobolomyces nakasei	-	-	-	CP727, CP735, CP741, CP751, CP756, CP762
Sporobolomyces carnicolor	-	CP799	-	CP707
**Subphylum Ustilaginomycotina**				
Anthracocystis elionuri	CP623	CP646, CP809, CP824	CP675, CP784	-
Dirkmeia churashimaensis	CP554, CP562, CP574, CP583, CP593, CP608, CP612, CP617	CP638, CP644, CP653, CP815	CP661, CP692, CP774, CP778	CP709
Moesziomyces antarcticus	CP556, CP564, CP624	-	CP671, CP681, CP684, CP697, CP770	CP726, CP745

Table S1. *Cont*

Taxa	Strain DMKU-			
	Suphan Buri	Chainat	Nakhon Sawan	Phetchabun
Ustilago trichophora	CP626	CP641	-	-
Ustilago siamensis	CP597	-	-	-
Ustilago sparsa	CP569, CP579, CP584, CP602, CP607, CP615	CP798, CP814	CP663, CP698, CP704	CP725, CP732

Table S2. Sequence similarity analysis of the D1/D2 region of corn phylloplane yeasts and their closely related species.

M13 profile	Strain with the same profile DMKU-	Representative strain DMKU-	GenBank accession no.	Closet specie (GenBank accession no.)	Nucleotide substitutions/ gap/total nt	% identity	Identification
1	CP563, CP635, CP669, CP705, CP791	CP563	LC430847	*Candida intermedia* (U44809)	2/1/475	99.37	*Candida intermedia*
		CP669	LC430848	*Candida intermedia* (U44809)	2/1/481	99.38	*Candida intermedia*
2	CP719	CP719	LC430849	*Candida quercitrusa* (U45831)	3/2/529	99.06	*Candida quercitrusa*
3	CP708, CP715, CP793	CP715	LC430850	*Candida saopaulonensis* (AY695398)	1/0/454	99.78	*Candida saopaulonensis*
		CP793	LC430851	*Candida saopaulonensis* (AY695398)	1/1/502	99.60	*Candida saopaulonensis*
4	CP802	CP802	LC430852	*Candida saopaulonensis* (AY695398)	1/0/456	99.78	*Candida saopaulonensis*
5	CP825	CP825	LC430853	*Candida uthaithanina* (AB588752)	1/0/543	99.82	*Candida uthaithanina*
6	CP628, CP666	CP628	LC430865	*Meyerozyma caribbica* (AY187283)	1/0/533	99.81	*Meyerozyma caribbica*
7	CP813, CP749, CP724	CP724	LC430864	*Meyerozyma caribbica* (AY187283)	1/0/533	99.81	*Meyerozyma caribbica*
8	CP679, CP710, CP767	CP679	LC430862	*Meyerozyma caribbica* (AY187283)	1/0/533	99.81	*Meyerozyma caribbica*
9	CP566	CP566	LC430858	*Meyerozyma caribbica* (AY187283)	0/0/558	100	*Meyerozyma caribbica*
10	CP629	CP629	LC430859	*Meyerozyma caribbica* (AY187283)	1/1/544	99.63	*Meyerozyma caribbica*
11	CP649, CP783	CP649	LC430860	*Meyerozyma caribbica* (AY187283)	2/0/510	99.61	*Meyerozyma caribbica*
12	CP670	CP670	LC430861	*Meyerozyma caribbica* (AY187283)	2/2/534	99.25	*Meyerozyma caribbica*
13	CP696	CP696	LC430863	*Meyerozyma caribbica* (AY187283)	2/0/521	99.62	*Meyerozyma caribbica*
14	CP586	CP586	LC430854	*Kodamaea ohmeri* (GU597323)	1/0/430	99.77	*Kodamaea ohmeri*
15	CP680, CP816	CP680	LC430855	*Metschnikowia lopburiensis* (AB697756)	0/0/406	100	*Metschnikowia lopburiensis*
		CP816	LC430857	*Metschnikowia lopburiensis* (AB697756)	0/0/417	100	*Metschnikowia lopburiensis*

Table S2. *Cont*

M13 profile	Strain with the same profile DMKU-	Representative strain DMKU-	GenBank accession no.	Closet specie (GenBank accession no.)	Nucleotide substitutions/ gap/total nt	% identity	Identification
16	CP722	CP722	LC430856	*Metschnikowia lopburiensis* (AB697756)	0/0/408	100	*Metschnikowia lopburiensis*
17	CP761, CP733, CP805	CP761	LC430868	*Pichia bruneiensis* (KY108804)	2/4/519	95.95	*Pichia bruneiensis*
18	CP737	CP737	LC430866	*Pichia bruneiensis* (KY108804)	1/3/504	96.03	*Pichia bruneiensis*
19	CP753	CP753	LC430867	*Pichia bruneiensis* (KY108804)	2/4/519	95.95	*Pichia bruneiensis*
20	CP826	CP826	LC430869	*Pichia bruneiensis* (KY108804)	3/1/498	95.98	*Pichia bruneiensis*
21	CP565	CP565	LC430870	*Pichia kudriavzevii* (EF550222)	0/0/522	100	*Pichia kudriavzevii*
22	CP672	CP672	LC430871	*Pichia kudriavzevii* (EF550222)	0/0/524	100	*Pichia kudriavzevii*
23	CP571, CP577	CP571	LC430873	*Wickerhamomyces anomalus* (U74592)	0/0/524	100	*Wickerhamomyces anomalus*
		CP577	LC430874	*Wickerhamomyces anomalus* (U74592)	0/0/544	100	*Wickerhamomyces anomalus*
24	CP657	CP657	LC430872	*Wickerhamomyces anomalus* (U74592)	2/0/534	99.63	*Wickerhamomyces anomalus*
25	CP555, CP647	CP555	LC428178	*Dioszegia zsoltii* (AF544245)	1/0582	99.83	*Dioszegia zsoltii*
		CP647	LC428179	*Dioszegia zsoltii* (AF544245)	1/0533	99.81	*Dioszegia zsoltii*
26	CP780	CP780	LC428180	*Dioszegia zsoltii* (AF544245)	1/0549	99.82	*Dioszegia zsoltii*
27	CP701	CP701	LC428181	*Hannaella oryzae* (AF075511)	0/0/530	100	*Hannaella oryzae*
28	CP775	CP775	LC428182	*Hannaella oryzae* (AF075511)	0/0/531	100	*Hannaella oryzae*
29	CP636	CP636	LC428183	*Hannaella pagnoccae* (FJ828959)	2/0557	99.64	*Hannaella pagnoccae*
30	CP723, CP810	CP810	LC428184	*Hannaella pagnoccae* (FJ828959)	0/0/550	100	*Hannaella pagnoccae*
31	CP812	CP812	LC428185	*Hannaella phetchabunensis* (AB922849)	0/0/543	100	*Hannaella phetchabunensis*
32	CP648, CP667	CP648	LC428186	*Hannaella phyllophila* (AB934929)	0/0/553	100	*Hannaella phyllophila*

Table S2. *Cont.*

M13 profile	Strain with the same profile DMKU-	Representative strain DMKU-	GenBank accession no.	Closet specie (GenBank accession no.)	Nucleotide substitutions/ gap/total nt	% identity	Identification
33	CP763	CP763	LC428187	*Hannaella phyllophila* (AB934929)	0/0/549	100	*Hannaella phyllophila*
34	CP631	CP631	LC428188	*Hannaella siamensis* (AB922844)	0/0/504	100	*Hannaella siamensis*
35	CP590, CP743, CP822	CP590<br>CP743	LC428189<br>LC428190	*Hannaella siamensis* (AB922844) *Hannaella siamensis* (AB922844)	0/0/561<br>0/0/516	100<br>100	*Hannaella siamensis*<br>*Hannaella siamensis*
36	CP721	CP721	LC428191	*Hannaella siamensis* (AB922844)	0/0/544	100	*Hannaella siamensis*
37	CP742	CP742	LC428201	*Hannaella sinensis* (AF189884)	0/0/538	100	*Hannaella sinensis*
38	CP568, CP606, CP610, CP630, CP637, CP643, CP652, CP655, CP665, CP668, CP703, CP712, CP716, CP728, CP765, CP803	CP568<br>CP652<br>CP728	LC428192<br>LC428193<br>LC428200	*Hannaella sinensis* (AF189884) *Hannaella sinensis* (AF189884)<br>*Hannaella sinensis* (AF189884)	0/0/578<br>0/0/526<br>0/0/516	100<br>100<br>100	*Hannaella sinensis*<br>*Hannaella sinensis*<br>*Hannaella sinensis*
39	CP787	CP787	LC428195	*Hannaella sinensis* (AF189884)	0/0/532	100	*Hannaella sinensis*
40	CP792	CP792	LC428196	*Hannaella sinensis* (AF189884)	0/0/532	100	*Hannaella sinensis*
41	CP819	CP819	LC428197	*Hannaella sinensis* (AF189884)	0/0/554	100	*Hannaella sinensis*
42	CP553, CP690	CP553<br>CP690	LC428198<br>LC428194	*Hannaella sinensis* (AF189884) *Hannaella sinensis* (AF189884)	0/0/578<br>1/0/518	100<br>99.81	*Hannaella sinensis*<br>*Hannaella sinensis*
43	CP595	CP595	LC428199	*Hannaella sinensis* (AF189884)	0/0/549	100	*Hannaella sinensis*
44	CP677	CP677	LC428202	*Hannaella sinensis* (AF189884)	0/1/534	99.81	*Hannaella sinensis*
45	CP576, CP796	CP576<br>CP796	LC370333<br>LC370334	*Vishniacozyma heimaeyensis* (DQ000317)<br>*Vishniacozyma heimaeyensis* (DQ000317)	10/1/562<br>10/1/562	98.04<br>98.04	*Vishiacozyma* aff. *heimaeyensis*<br>*Vishiacozyma* aff. *heimaeyensis*

Table S2. *Cont.*

M13 profile	Strain with the same profile DMKU-	Representative strain DMKU-	GenBank accession no.	Closet specie (GenBank accession no.)	Nucleotide substitutions/ gap/total nt	% identity	Identification
46	CP619	CP619	LC428203	*Kwoniella bestiolae* (FJ534903)	0/0/559	100	*Kwoniella bestiolae*
47	CP820	CP820	LC428204	*Kwoniella heveanensis* (AF075467)	1/0/557	99.82	*Kwoniella heveanensis*
48	CP611, CP634, CP760	CP611<br>CP760	LC428255<br>LC428259	*Papiliotrema flavescens* (AB035042)<br>*Papiliotrema flavescens* (AB035042)	1/0/511<br>0/0/526	99.81<br>100	*Papiliotrema flavescens*<br>*Papiliotrema flavescens*
49	CP642, CP700, CP720, CP734, CP740, CP786, CP808	CP786	LC428261	*Papiliotrema flavescens* (AB035042)	0/0/530	100	*Papiliotrema flavescens*
50	CP654, CP768	CP654<br>CP768	LC428256<br>LC428260	*Papiliotrema flavescens* (AB035042)<br>*Papiliotrema flavescens* (AB035042)	0/0/509<br>0/1/533	100<br>99.81	*Papiliotrema flavescens*<br>*Papiliotrema flavescens*
51	CP714	CP714	LC428257	*Papiliotrema flavescens* (AB035042)	0/0/511	100	*Papiliotrema flavescens*
52	CP585, CP660	CP585<br>CP660	LC428262<br>LC428266	*Papiliotrema laurentii* (AF075469)<br>*Papiliotrema laurentii* (AF075469)	1/0/551<br>556/557	99.82<br>99.82	*Papiliotrema laurentii*<br>*Papiliotrema laurentii*
53	CP560, CP567	CP560<br>CP567	LC428263<br>LC428264	*Papiliotrema laurentii* (AF075469)<br>*Papiliotrema laurentii* (AF075469)	1/0/551<br>513/514	99.82<br>99.81	*Papiliotrema laurentii*<br>*Papiliotrema laurentii*
54	CP797	CP797	LC428265	*Papiliotrema laurentii* (AF075469)	1/0/557	99.82	*Papiliotrema laurentii*
55	CP691, CP823, CP592	CP691<br>CP592	LC428268<br>LC428273	*Papiliotrema rajasthanensis* (AM262324)<br>*Papiliotrema rajasthanensis* (AM262324)	0/0/556<br>1/0/577	100<br>99.83	*Papiliotrema rajasthanensis*<br>*Papiliotrema rajasthanensis*
56	CP776	CP776	LC428269	*Papiliotrema rajasthanensis* (AM262324)	0/1/556	99.82	*Papiliotrema rajasthanensis*
57	CP603, CP754, CP790, CP800	CP603<br>CP790	LC428274<br>LC428270	*Papiliotrema rajasthanensis* (AM262324)<br>*Papiliotrema rajasthanensis* (AM262324)	0/0/560<br>0/0/564	100<br>100	*Papiliotrema rajasthanensis*<br>*Papiliotrema rajasthanensis*
58	CP552, CP580	CP552<br>CP580	LC428271<br>LC428272	*Papiliotrema rajasthanensis* (AM262324)<br>*Papiliotrema rajasthanensis* (AM262324)	1/0/577<br>0/0/577	99.83<br>100	*Papiliotrema rajasthanensis*<br>*Papiliotrema rajasthanensis*

Table S2. *Cont.*

M13 profile	Strain with the same profile DMKU-	Representative strain DMKU-	GenBank accession no.	Closet specie (GenBank accession no.)	Nucleotide substitutions/ gap/total nt	% identity	Identification
59	CP621	CP621	LC428267	*Papiliotrema rajasthanensis* (AM262324)	0/0/519	100	*Papiliotrema rajasthanensis*
60	CP781, CP818	CP781	LC428275	*Papiliotrema ruineniae* (LK023764)	0/1/533	99.81	*Papiliotrema ruineniae*
		CP818	LC428276	*Papiliotrema ruineniae* (LK023764)	0/0/539	100	*Papiliotrema ruineniae*
61	CP806	CP806	LC428277	*Papiliotrema ruineniae* (LK023764)	0/0/510	100	*Papiliotrema ruineniae*
62	CP801	CP801	LC370335	*Papiliotrema plantarum* (LC370335)	0/0/567	100	*Papiliotrema plantarum*
63	CP620	CP620	LC428278	*Saitozyma flava* (AF075497)	1/0/537	99.81	*Saitozyma flava*
64	CP758	CP758	LC428279	*Saitozyma flava* (AF075497)	1/0/545	99.82	*Saitozyma flava*
65	CP557	CP557	LC430175	*Cystobasidium calyptogenae* (AB025996)	0/0/563	100	*Cystobasidium calyptogenae*
66	CP558	CP558	LC430216	*Symmetrospora vermiculata* (AB279731)	0/0/563	100	*Symmetrospora vermiculata*
67	CP771	CP771	LC430217	*Symmetrospora vermiculata* (AB279731)	0/0/484	100	*Symmetrospora vermiculata*
68	CP821	CP821	LC430218	*Symmetrospora vermiculata* (AB279731)	0/0/542	100	*Symmetrospora vermiculata*
69	CP573	CP573	LC430182	*Rhodotorula mucilaginosa* (NG_055716)	1/0/555	99.82	*Rhodotorula mucilaginosa*
70	CP596	CP596	LC430181	*Rhodotorula mucilaginosa* (NG_055716)	0/1/539	99.81	*Rhodotorula mucilaginosa*
71	CP572	CP572	LC430183	*Rhodotorula paludigena* (AF070424)	0/0/556	100	*Rhodotorula paludigena*
72	CP604, CP640,	CP604	LC430185	*Rhodotorula paludigena* (AF070424)	0/0/476	100	*Rhodotorula paludigena*
	CP730, CP752,	CP640	LC430188	*Rhodotorula paludigena* (AF070424)	0/0/515	100	*Rhodotorula paludigena*
	CP746, CP757	CP730	LC430192	*Rhodotorula paludigena* (AF070424)	0/0/512	100	*Rhodotorula paludigena*
		CP752	LC430193	*Rhodotorula paludigena* (AF070424)	0/0/475	100	*Rhodotorula paludigena*
73	CP591	CP591	LC430184	*Rhodotorula paludigena* (AF070424)	0/0/558	100	*Rhodotorula paludigena*
74	CP614, CP650,	CP614	LC430186	*Rhodotorula paludigena* (AF070424)	0/0/521	100	*Rhodotorula paludigena*
	CP785	CP650	LC430190	*Rhodotorula paludigena* (AF070424)	0/0/525	100	*Rhodotorula paludigena*

Table S2. *Cont.*

M13 profile	Strain with the same profile DMKU-	Representative strain DMKU-	GenBank accession no.	Closet specie (GenBank accession no.)	Nucleotide substitutions/ gap/total nt	% identity	Identification
74		CP785	LC430195	*Rhodotorula paludigena* (AF070424)	0/0/525	100	*Rhodotorula paludigena*
75	CP625	CP625	LC430187	*Rhodotorula paludigena* (AF070424)	0/0/541	100	*Rhodotorula paludigena*
76	CP659	CP659	LC430191	*Rhodotorula paludigena* (AF070424)	0/0/525	100	*Rhodotorula paludigena*
77	CP645, CP693, CP702, CP777, CP794	CP645	LC430189	*Rhodotorula paludigena* (AF070424)	0/0/487	100	*Rhodotorula paludigena*
		CP702	LC430194	*Rhodotorula paludigena* (AF070424)	0/0/525	100	*Rhodotorula paludigena*
		CP794	LC430196	*Rhodotorula paludigena* (AF070424)	0/0/521	100	*Rhodotorula paludigena*
78	CP570, CP639, CP690, CP699	CP570	LC431632	*Rhodotorula toruloides* (AF070426)	5/0/581	99.14	*Rhodotorula* aff. *toruloides*
		CP639	LC431633	*Rhodotorula toruloides* (AF070426)	5/0/547	99.08	*Rhodotorula* aff. *toruloides*
		CP690	LC428194	*Rhodotorula toruloides* (AF070426)	1/0/518	99.81	*Rhodotorula toruloides*
		CP699	LC430199	*Rhodotorula toruloides* (AF070426)	1/0/488	99.81	*Rhodotorula toruloides*
79	CP589	CP589	LC430177	*Rhodosporidiobolus nylandii* (AF387123)	0/1/522	99.81	*Rhodosporidiobolus nylandii*
80	CP694, CP706, CP713	CP694	LC430178	*Rhodosporidiobolus ruineniae* (AF070434)	0/0/524	100	*Rhodosporidiobolus ruineniae*
		CP706	LC430179	*Rhodosporidiobolus ruineniae* (AF070434)	1/0/520	99.81	*Rhodosporidiobolus ruineniae*
		CP713	LC430180	*Rhodosporidiobolus ruineniae* (AF070434)	0/0/456	100	*Rhodosporidiobolus ruineniae*
81	CP658	CP658	LC430176	*Rhodosporidiobolus fluvialis* (AF070422)	1/0/539	99.81	*Rhodosporidiobolus fluvialis*
82	CP622	CP622	LC430215	*Rhodosporidiobolus odoratus* (AF387125)	1/0/509	99.80	*Rhodosporidiobolus odoratus*
83	CP551, CP601, CP683	CP551	LC430200	*Sporobolomyces blumeae* (AY213010)	0/0/530	100	*Sporobolomyces blumeae*
		CP601	LC430202	*Sporobolomyces blumeae* (AY213010)	0/0/496	100	*Sporobolomyces blumeae*
		CP683	LC430203	*Sporobolomyces blumeae* (AY213010)	0/1/487	99.79	*Sporobolomyces blumeae*
84	CP613, CP779	CP613	LC430205	*Sporobolomyces blumeae* (AY213010)	0/1/471	99.79	*Sporobolomyces blumeae*
		CP779	LC430206	*Sporobolomyces blumeae* (AY213010)	0/1/520	99.81	*Sporobolomyces blumeae*
85	CP575, CP605	CP575	LC430201	*Sporobolomyces blumeae* (AY213010)	0/0/530	100	*Sporobolomyces blumeae*
		CP605	LC430204	*Sporobolomyces blumeae* (AY213010)	0/1/492	99.81	*Sporobolomyces blumeae*

Table S2. *Cont.*

M13 profile	Strain with the same profile DMKU-	Representative strain DMKU-	GenBank accession no.	Closet specie (GenBank accession no.)	Nucleotide substitutions/ gap/total nt	% identity	Identification
86	CP741, CP751, CP762	CP741	LC430211	*Sporobolomyces nakasei* (KY109757)	1/0/511	99.81	*Sporobolomyces nakasei*
		CP751	LC430212	*Sporobolomyces nakasei* (KY109757)	1/0/510	99.81	*Sporobolomyces nakasei*
		CP762	LC430214	Sporobolomyces nakasei (KY109757)	0/0/511	100	*Sporobolomyces nakasei*
87	CP727, CP735, CP756	CP727	LC430209	*Sporobolomyces nakasei* (KY109757)	1/0/715	99.81	*Sporobolomyces nakasei*
		CP735	LC430210	*Sporobolomyces nakasei* (KY109757)	1/0/715	99.81	*Sporobolomyces nakasei*
		CP756	LC430213	*Sporobolomyces nakasei* (KY109757)	0/0/511	100	*Sporobolomyces nakasei*
88	CP799	CP799	LC430208	*Sporobolomyces carnicolor* (AY070008)	0/0/458	100	*Sporobolomyces carnicolor*
89	CP623, CP675, CP784, CP809	CP675	LC430821	*Anthracocystis elionuri* (AY740157)	1/0/544	99.82	*Anthracocystis elionuri*
		CP784	LC430822	*Anthracocystis elionuri* (AY740157)	0/0/558	100	*Anthracocystis elionuri*
		CP809	LC430823	*Anthracocystis elionuri* (AY740157)	0/0/555	100	*Anthracocystis elionuri*
90	CP646, CP824	CP646	LC430820	*Anthracocystis elionuri* (AY740157)	0/0/555	100	*Anthracocystis elionuri*
91	CP554, CP583, CP593, CP617, CP638, CP778	CP554	LC430825	*Dirkmeia churashimaensis* (AB548955)	0/0/531	100	*Dirkmeia churashimaensis*
		CP583	LC430829	*Dirkmeia churashimaensis* (AB548955)	0/0/420	100	*Dirkmeia churashimaensis*
		CP593	LC430830	*Dirkmeia churashimaensis* (AB548955)	0/0/549	100	*Dirkmeia churashimaensis*
		CP709	LC430833	*Dirkmeia churashimaensis* (AB548955)	0/0/566	100	*Dirkmeia churashimaensis*
92	CP562, CP608, CP774	CP562	LC430826	*Dirkmeia churashimaensis* (AB548955)	0/0/531	100	*Dirkmeia churashimaensis*
		CP608	LC430831	*Dirkmeia churashimaensis* (AB548955)	0/0/551	100	*Dirkmeia churashimaensis*
93	CP574, CP644, CP653, CP692	CP574	LC430827	*Dirkmeia churashimaensis* (AB548955)	0/0/531	100	*Dirkmeia churashimaensis*
94	CP612, CP661, CP815	CP612	LC430832	*Dirkmeia churashimaensis* (AB548955)	0/0/540	100	*Dirkmeia churashimaensis*
		CP815	LC430828	*Dirkmeia churashimaensis* (AB548955)	0/0/556	100	*Dirkmeia churashimaensis*
95	CP556, CP681, CP684	CP681	LC430834	*Moesziomyces antarcticus* (JN940521)	0/0/545	100	*Moesziomyces antarcticus*
		CP684	LC430835	*Moesziomyces antarcticus* (JN940521)	0/0/544	100	*Moesziomyces antarcticus*

Table S2. *Cont.*

M13 profile	Strain with the same profile DMKU-	Representative strain DMKU-	GenBank accession no.	Closet specie (GenBank accession no.)	Nucleotide substitutions/ gap/total nt	% identity	Identification
96	CP564, CP624, CP671, CP697, CP745, CP726, CP770	CP697	LC430836	*Moesziomyces antarcticus* (JN940521)	0/0/545	100	*Moesziomyces antarcticus*
97	CP626, CP641	CP626	LC430837	*Ustilago trichophora* (AY740125)	0/0/567	100	*Ustilago trichophora*
		CP641	LC430838	*Ustilago trichophora* (AY740125)	0/0/505	100	*Ustilago trichophora*
98	CP597	CP597	LC430839	*Ustilago siamensis* (AB117963)	0/1/536	99.81	*Ustilago siamensis*
99	CP569, CP579, CP607, CP615, CP663, CP798	CP569	LC430840	*Ustilago sparsa* (JN367335)	0/0/529	100	
		CP579	LC430845	*Ustilago sparsa* (JN367335)	0/0/490	100	*Ustilago sparsa*
		CP663	LC430846	*Ustilago sparsa* (JN367335)	2/0/556	99.64	*Ustilago sparsa*
		CP798	LC430844	*Ustilago sparsa* (JN367335)	1/0/559	99.82	*Ustilago sparsa*
							Ustilago sparsa
100	CP584, CP704, CP814 CP732	CP584	LC430841	*Ustilago sparsa* (JN367335)	0/0/494	100	*Ustilago sparsa*
		CP732	LC430843		0/0/550	100	*Ustilago sparsa*
101	CP602, CP698, CP725	CP698	LC430842	*Ustilago sparsa* (JN367335)	0/0/529	100	*Ustilago sparsa*

Table S3. Sequence similarity analysis of the ITS region of corn phylloplane isolates showing ≥ 2 nucleotide substitutions in the D1/D2 region

Isolate	GenBank accession no. of D1/D2, ITS region	Closest species	Nucleotide substitutions/ gap/total nt		Identification
			D1/D2 region	ITS region	
DMKU-CP570	LC431632, LC431624	*Rhodotorula toruloides* CBS 349 (AF070426, AF444489)	5/0/581	5/4/584	Potential new species closest to *Rhodotorula toruloides*
DMKU-CP639	LC431633, LC431626	*Rhodotorula toruloides* CBS 349 (AF070426, AF444489)	5/0/547	5/4/584	Potential new species closest to *Rhodotorula toruloides*
DMKU-CP576	LC370333, LC431625	*Vishniacozyma heimaeyensis* CBS 8933^T (DQ000317, KF036591)	10/1/562	19/11/492	Potential new species closest to *Vishniacozyma heimaeyensis*
DMKU-CP796	LC370334, LC431630	*Vishniacozyma heimaeyensis* CBS 8933^T (DQ000317, KF036591)	10/1/562	19/11/492	Potential new species closest to *Vishniacozyma heimaeyensis*

Table S4. Similarity coefficient between yeast communities in pairwise comparisons based on the Jaccard similarity index.

	**S1**	**S2**	**S3**	**S4**	**S5**	**S6**	**S7**	**S8**	**S9**	**S10**	**S11**	**S12**	**S13**	**S14**	**S15**
**S1**	1.00														
**S2**	0.32	1.00													
**S3**	0.29	0.40	1.00												
**S4**	0.29	0.24	0.36	1.00											
**S5**	0.22	0.23	0.33	0.25	1.00										
**S6**	0.24	0.20	0.28	0.22	0.32	1.00									
**S7**	0.12	0.13	0.22	0.00	0.13	0.11	1.00								
**S8**	0.18	0.32	0.38	0.15	0.20	0.23	0.15	1.00							
**S9**	0.47	0.29	0.33	0.39	0.44	0.21	0.06	0.20	1.00						
**S10**	0.35	0.24	0.46	0.33	0.32	0.27	0.14	0.28	0.39	1.00					
**S11**	0.37	0.19	0.29	0.44	0.26	0.29	0.00	0.10	0.33	0.35	1.00				
**S12**	0.18	0.19	0.50	0.13	0.20	0.31	0.29	0.33	0.20	0.42	0.14	1.00			
**S13**	0.15	0.10	0.25	0.11	0.24	0.14	0.20	0.27	0.17	0.33	0.12	0.18	1.00		
**S14**	0.18	0.19	0.29	0.21	0.50	0.29	0.15	0.29	0.33	0.35	0.16	0.23	0.19	1.00	
**S15**	0.18	0.19	0.29	0.28	0.33	0.29	0.15	0.29	0.26	0.35	0.22	0.33	0.19	0.57	1.00
